# Mechanisms and treatment of atrial fibrillation and stroke

**DOI:** 10.1515/jtim-2025-0024

**Published:** 2025-07-30

**Authors:** Shuai Wang, Zhi Liu

**Affiliations:** Department of Emergency, Xuanwu Hospital, Capital Medical University, Beijing 100053, China

Atrial fibrillation (AF) is a prevalent cardiac arrhythmia that is observed to increase in frequency with advancing age. In addition to the natural process of aging, a number of chronic health conditions have been associated with the occurrence and increased risk of developing AF. Such conditions include hypertension, diabetes, congestive heart failure, coronary artery disease and valvular heart disease, among others.

AF represents the most prevalent etiology of cardioembolic stroke, contributing to approximately 25% of all ischemic stroke cases. Furthermore, it is associated with elevated rates of stroke severity and mortality among affected individuals. In the aftermath of an acute ischemic stroke or transient ischemic attack (TIA), AF is the most frequently observed cardiac arrhythmia, identified in up to 75% of cases through electrocardiographic changes.^[1]^

AF is associated with an increased risk of stroke due to the presence of atrial contractility, which is weakened as a result of the condition. This leads to disorganized myocyte function, blood stasis and an elevated likelihood of thromboembolism. Furthermore, longer episodes of AF can also cause damage to the cellular structure of atrial myocytes, including the contractile apparatus, cellular organelles or cause cellular death. Prothrombogenic factors are expressed at the surface of endothelial cells, resulting in the adhesion of platelets and leucocytes to the atrial endocardium, particularly in the left atrial appendage. This initiates the formation of thrombin.^[2]^ This process revealed the involvement of multiple molecular pathways in the pathogenesis of myocardial alterations and thrombogenic atrial changes. Notably, inflammatory path ways have been identified as a key mechanism underlying atrial thrombogenesis. It has been proposed that oxidative stress plays a crucial role in atrial electrical and structural remodeling in AF. There is evidence to suggest that reactive oxygen and nitrogen species (ROS/RNS) and redox signaling pathways are central to the process of atrial remodeling.^[3]^ A positive correlation was demonstrated between CRP and interleukin-6 and left atrial diameter. Elevated levels of these biomarkers were found to increase the risk of vascular death and thromboembolic events.^[4]^ The interplay between AF’s impact on atrial contractility and other factors together contribute to the formation of thrombi that can cause stroke.^[5]^ Mult territorial embolisms are more frequently observed in cases of cardioembolic stroke resulting from atrial fibrillation. Additionally, atrial fibrillation has been linked to the occurrence of silent cerebral infarctions (SCI) and TIA. The pathophysiology of stroke patients with new-onset atrial fibrillation may involve in the stroke-heart crosstalk, including the hypothalamic-pituitary-adrenal axis (HPA), inflammation, atherosclerosis, the microbiota-immune axis, and the neurohumoral system.^[6]^

**Figure 1:**
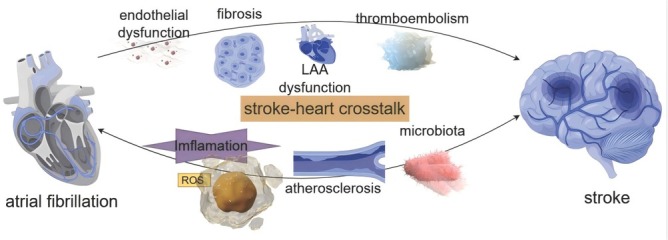
Key pathways and mechanisms in atrial fbrillation and stroke.

AF is an important cause in patients presenting with an embolic stroke of undetermined source (ESUS). Especially with advanced age, left atrium enlargement and frequent supraventricular extrasystoles.^[7,8]^ Despite limited evidence supporting AF screening for primary prevention of cardioembolic stroke in the general population, ECG screening is crucial, and while smartphones and smartwatches offer potential for AF screening and monitoring, further research is needed to establish their appropriate use and target populations.^[9]^ Figure 1 illustrates the potential mechanisms linking atrial fibrillation to stroke.

For AF without stroke, Rhythm control is recommended for patients with symptomatic AF according to the current guidelines from the European Society of Cardiology.^[10]^ Early rhythm control within 1 year after AF diagnosis might be beneficial to prevent recurrent stroke in patients with incident AF and a history of stroke. In select patients presenting with symptomatic paroxysmal atrial fibrillation, catheter ablation should be considered as a primary rhythm control strategy.^[10]^ However, it encounters two primary challenges: firstly, the success rate falls short of being optimal and remains relatively stagnant; secondly, there exists a risk of infrequent yet potentially severe complications and mortality. For AF with stroke, thrombolytic therapy within 4.5 hours improves the prognosis of ischemic stroke. For patients with valvular atrial fibrillation, or anticoagulation with vitamin K antagonists is recommended for stroke prevention. Whereas for those with non-valvular AF, direct oral anticoagulants are globally preferred by AF guidelines for anticoagulant therapy to prevent strokes.^[10]^ But it is unclear when to (re-) start oral anticoagulation (OAC) after an acute ischemic stroke in patients with AF. Initiating OAC for the majority of patients experiencing an acute ischemic stroke associated with AF is considered appropriate between 4 to 14 days post the onset of neurological symptoms. Anticoagulation should be individualized according to CHA2DS2-VASc score versus HAS-BLED score.^[10]^ Left atrial appendage (LAA) occlusion represents a viable alternative for patients who are at elevated risk of stroke and for whom OACs are contraindicated.^[1]^

In conclusion, AF is the most common tachyarrhythmia and a major cause of silent and clinical stroke. The relationship between AF and cerebral ischemia is bidirectional and we now have a better understanding of the pathophysiology of thrombosis and other mechanisms associated with AF. Early diagnosis of atrial fibrillation is important because of the early prevention of stroke. Individualized treatment for AF patients with/without stroke is necessary.
